# Recanalization therapy in stroke patients with malignancies: in-hospital outcomes by cancer subtype in a nationwide administrative data analysis

**DOI:** 10.1007/s00415-026-13851-9

**Published:** 2026-05-29

**Authors:** Robin Jansen, Sarah Gammersbach, Tristan Kölsche, Sven G. Meuth, Antje Schmidt-Pogoda, Stefanie Schreiber, Tobias Ruck, John-Ih Lee, Michael Gliem

**Affiliations:** 1 Department of Neurology, Medical Faculty, Heinrich-Heine-University, Moorenstr. 5, 40225 Düsseldorf, Germany; 2 Department of Neurology, Medical Faculty, Otto Von Guericke University, Magdeburg, Germany; 3 Department of Neurology with Institute of Translational Neurology, University of Münster, Münster, Germany; 4 Department of Neurology with Heimer Institute for Muscle Research, University Hospital Bergmannsheil, Bochum, Germany

**Keywords:** Ischemic stroke, acute therapy, cancer, thrombolysis, thrombectomy, outcome

## Abstract

**Objective:**

Stroke is a common risk among patients with cancer, and the odds of a fatal stroke are estimated to be twice those of the general population. Due to the exclusion of cancer patients from major intravenous thrombolysis and thrombectomy trials, existing literature consists largely of small case series lacking sufficient statistical power. We aimed to evaluate the safety profile of acute stroke therapies in patients with active cancer, stratified by cancer localization, using a nationwide administrative dataset.

**Methods:**

This retrospective, large-scale cohort study utilized data from the German Federal Statistical Office (DESTATIS). We analyzed all inpatient cases of acute ischemic stroke receiving recanalization therapy (intravenous thrombolysis and endovascular thrombectomy) in Germany. The primary endpoints were in-hospital mortality and safety outcomes (intracranial bleeding, subarachnoid hemorrhage, and acute anemia). To control for baseline characteristics, adjusted odds ratios (aOR) were calculated using multivariable logistic regression.

**Results:**

We analyzed 154,333 patients receiving intravenous thrombolysis (2482 with and 151,851 without cancer) and 39,534 receiving endovascular thrombectomy (1580 with and 37,954 without cancer). In the thrombolysis cohort, patients with cancer had significantly higher rates of in-hospital death (10.88% vs. 6.26%; OR 1.83; *p* < 0.001) and intracranial bleeding (5.76% vs. 4.58%; OR 1.27; *p* = 0.005). Similarly, in the thrombectomy cohort, active cancer was associated with increased in-hospital mortality (28.10% vs. 20.00%; OR 1.56; *p* < 0.001) and subarachnoid hemorrhage (6.14% vs. 4.67%; OR 1.33; *p* = 0.007). After multivariable adjustment, stratification revealed highly heterogeneous complication profiles depending heavily on the specific cancer subtype.

**Conclusions:**

This large-scale analysis demonstrates that while mortality and specific bleeding risks are generally elevated in stroke patients with malignancies, these risks depend strongly on the cancer subtype. These findings advocate for a tailored, risk-based approach to recanalization therapy, weighing individual complication profiles rather than a general exclusion of patients with cancer from acute stroke therapies.

**Supplementary Information:**

The online version contains supplementary material available at https://doi.org/10.1007/s00415-026-13851-9.

## Introduction

With 6.5 million deaths globally in 2019, stroke is the second-leading cause of death behind ischemic heart disease [1]. In the United States, every 40 s someone has a stroke and every 4 min someone dies from stroke [2]. An estimated 50 million people worldwide are living with a cancer diagnosis [3]. From large epidemiological studies showed a risk of fatal stroke in cancer patients of more than twice that of the general population, with an increasing risk in subsequent years. At the highest risk were patients with gastrointestinal malignancies (especially of the pancreas, liver, and esophagus), and brain malignancies [4]. Recent data from the same German nationwide dataset used in this study confirmed "cancer" as a strong independent predictor of in-hospital mortality in stroke patients, with an adjusted odds ratio (OR) of 2.11 [5]. To complement this established epidemiological evidence and deepen the analysis regarding the safety of revascularization, we investigated specific complication profiles across different cancer entities [5].

The etiology of stroke in cancer patients is unclear; however, a hypercoagulative state in cancer patients is highly suggestive [6]. Stroke in cancer can also be caused by cancer treatments such as radiation, cisplatin, methotrexate or antibody therapy such as bevacizumab [6].

Today, the only acute therapeutic options are intravenous thrombolysis within 4.5 h and in selected patients, up to 9 h from stroke onset or catheter-guided endovascular thrombectomy within 6 up to 24 h after stroke onset [7]. Much of the existing literature on stroke acute therapy and cancer patients consists of small case series, single-center reports, or analyses focusing on specific pathophysiological mechanisms derived from individual cases. A substudy from the MR CLEAN registry, for example, with 124 patients which underwent endovascular thrombectomy and malignancies, showed a worse 3-month-mRS (mRS score 0–2: 22.6% vs 42.0%, adjusted OR [aOR] 0.5, 95% CI 0.3–0.8) and more deaths in patients with active malignancy compared to patients without cancer (52.2% vs 26.5%, aOR 3.2, 95% CI 2.1–4.9) [8]. Recent data on thrombolysis in cancer patients, e.g., in the 2025 published ITACA-Stroke study showed beneficial effects of acute therapy compared to no therapy in cancer patients (OR: 2.56, 95% CI 1.45–4.52, *p* = 0.001) [9]. However, analysis of the association between cancer site and secondary outcomes was not performed. Due to the restricted sample size, the association between specific cancer sites and secondary outcomes was limited in these studies. Randomized controlled trials (RCTs) addressing this specific population are difficult to realize due to the ethical complexity of withholding potentially lifesaving therapy and the heterogeneity of cancer types. To bridge this gap and complement the data from smaller cohorts, we performed a large-scale analysis using administrative data. This approach allows us to stratify risks for different cancer categories with a high number of patients, albeit with the limitations inherent to retrospective administrative datasets.

In Germany, there are approximately 200,000 strokes per year, which are recorded in the case-based hospital disease related groups (DRG) statistics. This complete annual record of all inpatient hospital cases serves as the basis for the flat rate per case system that was implemented in 2003. By using all DRG codes of patient cases reported to the federal bureau of statistics (deutsches statistsches Bundesamt; DESTATIS) we analyze mortality and complications of patients with stroke and acute therapy. The objective of this study is therefore to analyze a comprehensive administrative dataset (DRG data) to identify specific tumor entities associated with complications following thrombolysis and endovascular thrombectomy. By identifying these high-risk subgroups, we aim to provide data that can inform the development of refined clinical protocols and specialized management strategies for this vulnerable patient population.

## Materials and methods

### Study population

The German Federal Statistical Offices (DRG-Statistic, destatis.de) provided the analyzed datasets. German hospitals report all in-hospital cases in the format of ICD-Codes (International Classification of Diseases; *ICD*) in the 10th version and German modification as well as all procedures such as thrombectomy or thrombolysis encoded by an operating and procedures key system (OPS-Code) to the Federal Statistics office. Further information is available under digital object identifier: https://doi.org/10.21242/23141.2019.00.00.2.1.1. In Germany, hospital reimbursement is based on the Diagnosis Related Groups (DRG) system, implemented in 2003. This system requires a complete annual record of all inpatient cases to be reported to the Federal Bureau of Statistics (DESTATIS). The coding quality is generally high because it directly determines hospital reimbursement; diagnoses and procedures that trigger higher reimbursement groups (such as stroke, thrombolysis, thrombectomy, and intensive care treatments) are subject to rigorous external review by insurance companies (MDK). Consequently, complications relevant to reimbursement (e.g., intracranial hemorrhage, need for transfusion) are typically well-documented. However, clinical parameters that do not influence the DRG grouping (such as NIHSS scores or specific laboratory values) are not part of this dataset.

Admission, use and data security are closely supervised and regulated by the office itself and the German Coding Guidelines. The presented analysis therefore is case- and not patient-based. In the present study we used the code I63 for ischemic stroke (first or recurrent) in combination with intravenous thrombolysis (OPS code 8-020.8) and for mechanical thrombectomy (OPS code 8-836.80). Low numbers of patients with a distinct coding pattern caused a missing value due to data protection rules.

The local ethics committee of the Heinrich-Heine University Duesseldorf approved the project and study number 2022-2251 in accordance with the STROBE and RECORD guidelines. Due to our registry-only, fully anonymous data approach, no participant consent was needed.

### Statistical analysis

Results are reported as absolute numbers and percentages. Associations between all categorical variables were analyzed using the Chi-Square test or Fisher’s exact test where appropriate. As normality assumptions were violated by Shapiro–Wilk test, group differences were assessed using non-parametric Wilcoxon–Mann–Whitney U test (for independent data). Null hypothesis (H0) was defined as no difference between groups. *p* values < 0,05 were considered as statistically significant.

To account for significant baseline imbalances between cancer and non-cancer cohorts, multivariable logistic regression models were applied. We calculated adjusted odds ratios (aOR) and corresponding 95% confidence intervals (CI) to assess the association between specific cancer subtypes and clinical outcomes (death, intracranial bleeding, acute anemia and subarachnoid hemorrhage). All presented odds ratios were adjusted for: age, sex, atrial fibrillation, diabetes mellitus, arterial hypertension, hyperlipidemia, nicotine abuse and coronary artery disease. In multivariable logistic regression models, subgroups with zero events (complete separation) were omitted from the respective subgroup analyses as adjusted odds ratios could not be reliably estimated. Results are presented as forest plots with a logarithmic x-axis scale and heatmaps. For statistical analysis STATA Version 18 (StataCorp LP, College Station, TX) and Microsoft R (Version 4.5.2) were used. All data are available from the corresponding authors by reasonable request.

## Results

### Study population

**Fig. 1:**
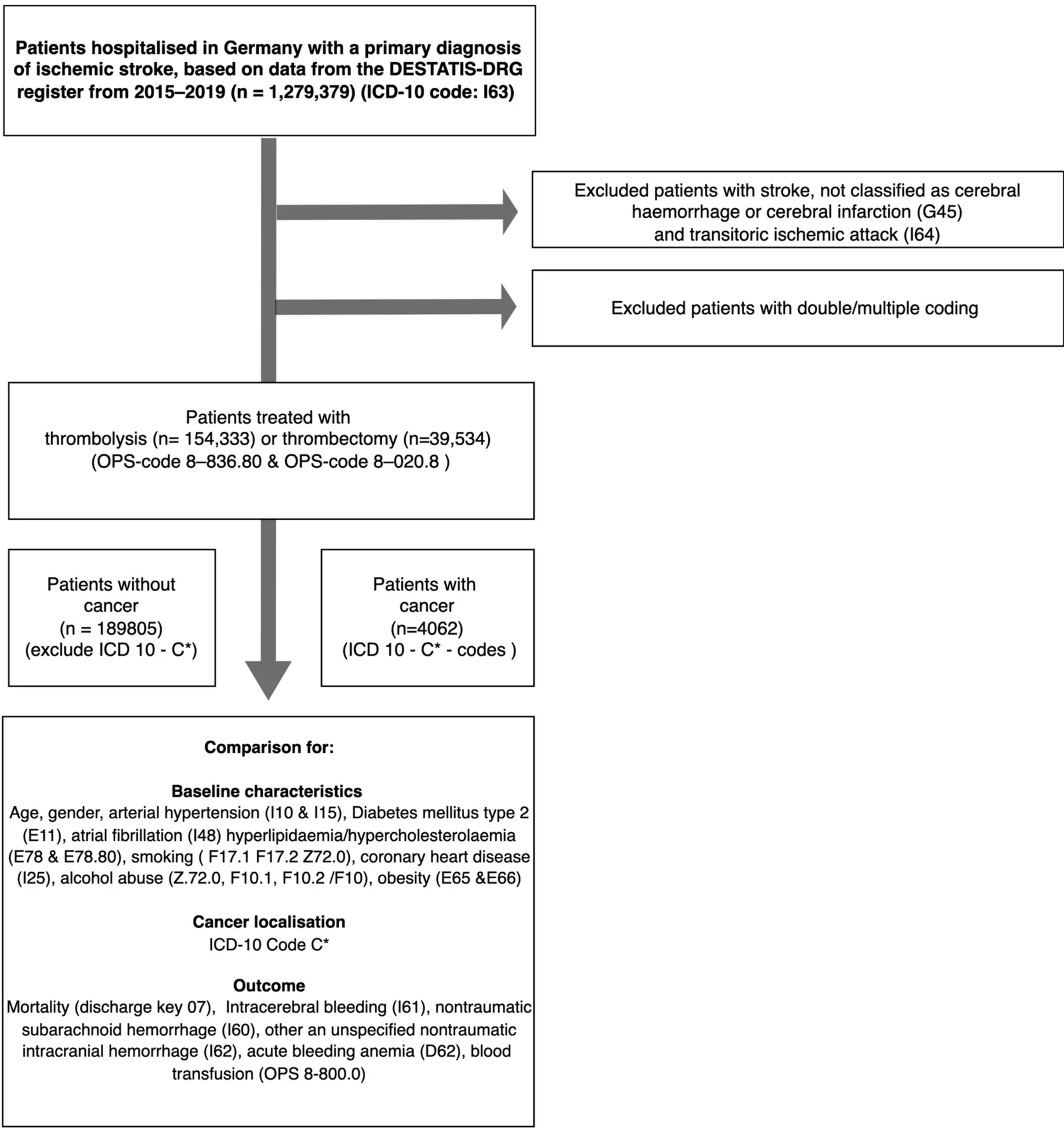
Flowchart of the DRG-code-based patient selection process to identify the final study cohort of ischemic stroke patients treated with thrombolysis or thrombectomy, stratified by cancer status. *CHD* coronary heart disease, *DESTATIS-DR*G Federal statistical office of Germany Statistisches Bundesamt), *ICD-10*, International Statistical Classification of Diseases and Related Health Problems, 10th revision, *n* number of patients, *OPS* Operationen- und Prozedurenschlüssel (German procedure classification)

Baseline characteristics for patients who suffered a stroke (coded as ICD-10 I63.-) and underwent thrombolysis (*n* = 154,333, procedure code 8–020.8) or thrombectomy (*n* = 39,534, procedure code 8-836.8) with cancer status (coded within ICD-10 C00-C97) are shown in Table 1. Patients with cancer who underwent thrombolysis had a higher proportion of males (58.22% vs. 52.68%, *p* < 0.001), a significantly higher prevalence of diabetes mellitus type 2 (26.31% vs. 23.44%, *p* = 0.001), atrial fibrillation (29.73% vs. 25.04%, *p* < 0.001), and coronary heart disease (17.16% vs. 14.17%, *p* < 0.001). Cancer patients had a significantly lower prevalence of arterial hypertension (66.11% vs. 68.96%, *p* = 0.002) and hyperlipidemia (37.63% vs. 41.91%, *p* < 0.001). Patients with cancer receiving thrombectomy (*n* = 1580) were significantly more likely to be male (50.44% vs. 45.96%, *p* < 0.001) and had a significantly lower prevalence of nearly all major cardiovascular risk factors: arterial hypertension (55.76% vs. 65.28%, *p* < 0.001), diabetes mellitus type 2 (18.61% vs. 22.08%, *p* = 0.001), atrial fibrillation (38.41% vs. 48.89%, *p* < 0.001), hyperlipidemia (25.19% vs. 29.51%, *p* < 0.001), and obesity (2.72% vs. 4.13%, *p* = 0.006) (Fig. 1).

**Table 1:** Baseline characteristics for patients who underwent thrombolysis, thrombectomy and with or without cancer in total numbers and proportion in percent as well as *p* values, statistical differences were assessed via Chi-Square and for age via Wilcoxon-Rank-Test

Thrombolysis *N* = 154,333			
	Without cancer *N* = 151,851	With cancer *N* = 2482	
Sex			
Female (%)	71,850 (47.31)	1037 (41.78)	< 0.001
Male (%)	79,996 (52.68)	1445 (58.22)	< 0.001
Other (%)	5 (0.003)	0	< 0.001
Arterial hypertension (%)	104,721 (68.96)	1641 (66.11)	0.002
Diabetes mellitus type 2 (%)	35,596 (23.44)	653 (26.31)	0.001
Atrial fibrillation (%)	38,028 (25.04)	738 (29.73)	< 0.001
Hyperlipidemia (%)	63,644 (41.91)	934 (37.63)	< 0.001
Nicotine abuse (%)	6619 (4.36)	105 (4.23)	0.756
Coronary heart disease (%)	21,513 (14.17)	426 (17.16)	< 0.001
Obesity (%)	6661 (4.39)	95 (3.83)	0.177
Alcohol abuse (%)	3408 (2.24)	52 (2.10)	0.626
Thrombectomy *N* = 39,534			
	Without cancer *N* = 37954	With cancer *N* = 1580	*p* value
Sex			
Female (%)	20,512 (54.04)	783 (49.56)	< 0.001
Male (%)	17,442 (45.96)	797 (50.44)	< 0.001
Arterial hypertension (%)	24,776 (65.28)	881 (55.76)	< 0.001
Diabetes mellitus type 2 (%)	8380 (22.08)	294 (18.61)	0.001
Atrial fibrillation (%)	18,555 (48.89)	607 (38.41)	< 0.001
Hyperlipidemia (%)	11,199 (29.51)	398 (25.19)	< 0.001
Nicotine abuse (%)	1454 (3.83)	64 (4.05)	0.656
Coronary heart disease (%)	5884 (15.50)	237 (15.00)	0.588
Obesity (%)	1567 (4.13)	43 (2.72)	0.006
Alcohol abuse (%)	844 (2.22)	29 (1.84)	0.303

### Outcome of patients with acute therapy with and without malignancies

Outcomes of patients who suffered a stroke with and without cancer are shown in Table 2. In the thrombolysis cohort, patients with cancer experienced significantly higher in-hospital mortality (10.88% vs 6.26%; OR 1.83, *p* < 0.001, discharge key 07). Regarding key safety outcomes, the incidence of intracranial bleeding (ICD-10 I61.- or I62.-) was significantly elevated in the cancer cohort (5.76% vs. 4.58%; OR 1.27, *p* = 0.005) and cancer patients suffered significantly more frequent acute anemia (3.99% vs. 0.91%; *p* < 0.001, ICD-10 D62).

**Table 2:** Outcome of patients with and without cancer in dependence of acute therapy, total numbers and proportion in percent, significance *p* < 0.001 via Chi-Square

Thrombolysis *N* = 154333			
	Without malignoma *N* = 151,851	With malignoma *N* = 2482	*p* value
Death (%)	9503 (6.26)	270 (10.88)	< 0.001
Intracranial bleeding (%)	6956 (4.58)	143 (5.76)	0.005
Subarachnoid hemorrhage (%)	1229 (0.81)	25 (1.01)	0.275
Hypovolemic shock (%)	142 (0.09)	8 (0.32)	< 0.001
Shock (%)	88 (0.06)	0 (0.0)	0.23
Acute anemia (%)	1375 (0.91)	99 (3.99)	< 0.001

Patients with cancer experienced significantly higher in-hospital mortality (10.88% vs. 6.26%; *p* < 0.001, discharge key 07). Regarding safety outcomes, the incidence of intracranial bleeding (ICD-10 I61.- or I62.-) was significantly elevated in the cancer cohort (5.76% vs. 4.58%; *p* = 0.005) and cancer patients suffered significantly more frequent acute anemia (3.99% vs. 0.91%; *p* < 0.001, ICD-10 D62). Hypovolemic shock was also significantly more prevalent among cancer patients (0.32% vs. 0.09%; *p* < 0.001, ICD-10 R57.1).

Table 3 shows the complication pattern of 39,534 stroke patients treated with thrombectomy by cancer status. Patients with cancer had a significantly higher in-hospital mortality rate (28.10% vs. 20.00%; OR 1.56, *p* < 0.001) and a significantly higher incidence of subarachnoid hemorrhage (6.14% vs. 4.67%; OR 1.33, *p* = 0.007, ICD-10 I60.-) and acute anemia (8.35% vs. 4.49%; *p* < 0.001, ICD-10 D62). However, no significant differences were found between the groups for the rates of overall intracranial bleeding (8.16% vs. 8.11%; *p* = 0.941), hypovolemic shock (ICD-10 R57.1) (0.25% vs. 0.35%; *p* = 0.529), or decompressive craniectomy (2.15% vs. 2.98%; *p* = 0.056, OPS 5-012.f). It is important to note that administrative data capture all-cause in-hospital mortality, which inherently conflates procedure-related complications with terminal cancer progression.

**Table 3:** Outcome of patients with and without cancer depending on acute therapy, total numbers and proportion in percent, significance *p* < 0.001 via Chi-Square

Thrombectomy *N* = 39534			
	Without malignoma *N* = 37954	With malignoma *N* = 1580	*p* value
Death (%)	7589 (20.00)	444 (28.10)	< 0.001
Intracranial bleeding (%)	3079 (8.11)	129 (8.16)	0.941
Subarachnoid hemorrhage (%)	1774 (4.67)	97 (6.14)	0.007
Hypovolemic shock (%)	132 (0.35)	4 (0.25)	0.529
Acute anemia (%)	1705 (4.49)	132 (8.35)	< 0.001
Craniectomy (%)	1132 (2.98)	34 (2.15)	0.056

### Outcome of patients with acute therapy and cancer localization

**Fig. 2:**
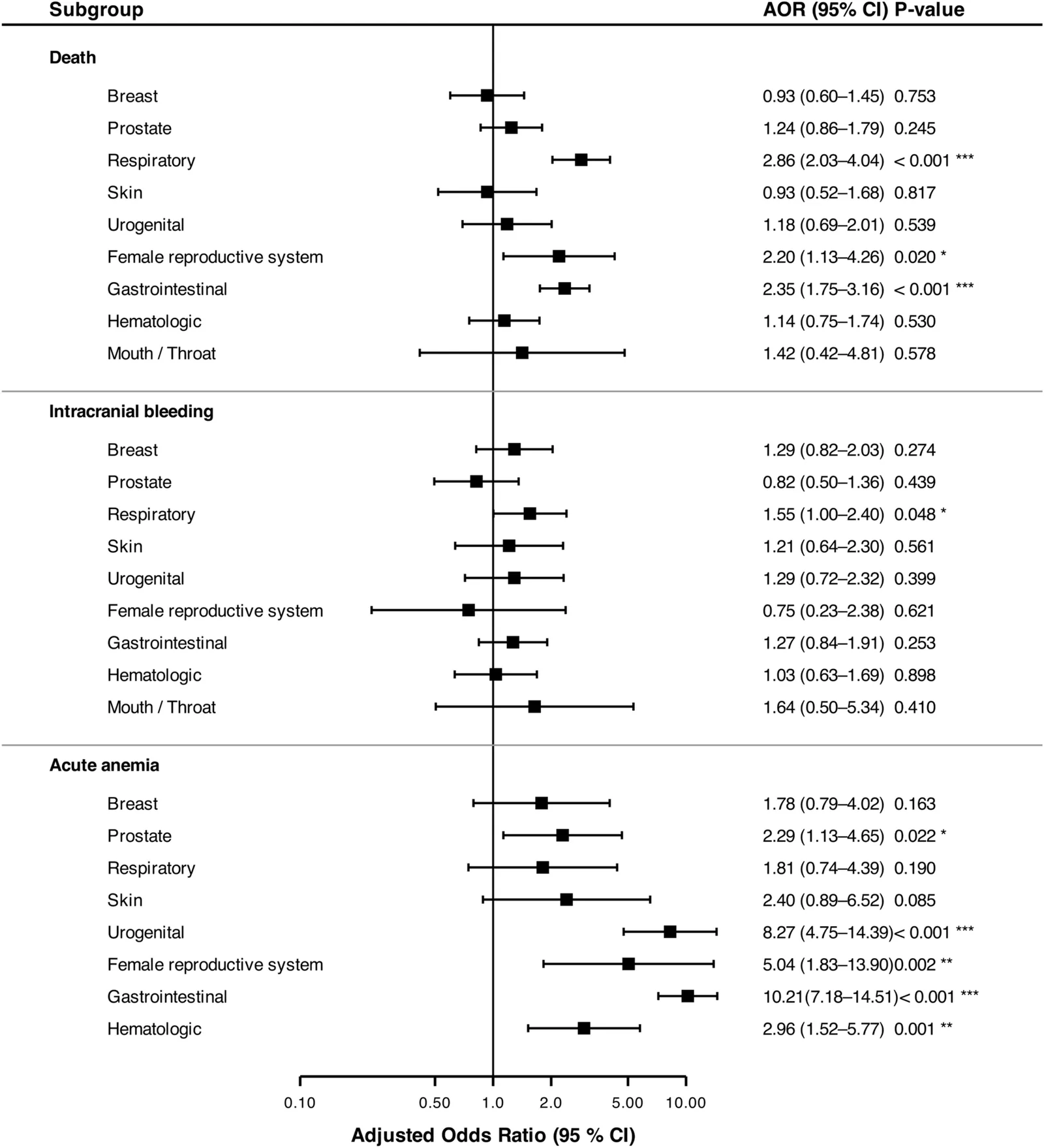
Association between specific cancer subtypes and in-hospital complications following intravenous thrombolysis. Forest plots display adjusted Odds Ratios (aOR) and corresponding 95% Confidence Intervals (CI) compared to the non-cancer control group. Estimates were derived from multivariable logistic regression models adjusting for age, sex, atrial fibrillation, diabetes mellitus, arterial hypertension, hyperlipidemia, nicotine abuse and coronary artery disease. The vertical reference line represents an aOR of 1.0 (indicating no difference in risk)

In the thrombolysis cohort, patients with respiratory cancer showed the highest adjusted odds of death (aOR 2.86, 95%CI 2.03–4.04), closely followed by gastrointestinal cancer (aOR 2.35, 95%CI 1.75–3.16), whereas patients with breast cancer and skin cancer presented the lowest relative risk (aOR 0.93 for both) (Fig. 2).

**Fig. 3:**
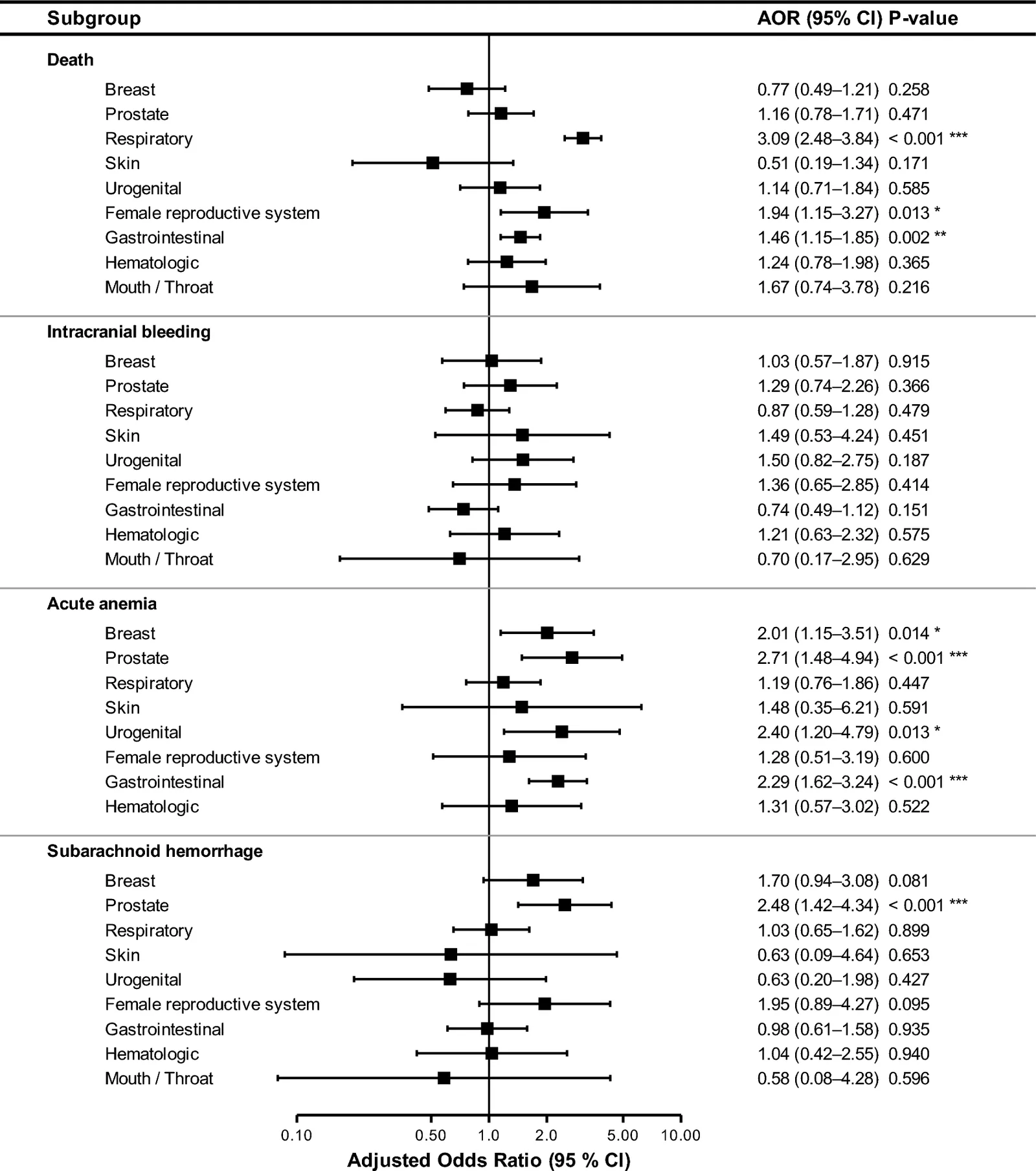
Association between specific cancer subtypes and in-hospital complications following endovascular thrombectomy. Forest plots display adjusted Odds Ratios (aOR) and corresponding 95% confidence intervals (CI) compared to the non-cancer control group. Estimates were derived from multivariable logistic regression models adjusting for age, sex, atrial fibrillation, diabetes mellitus, arterial hypertension, hyperlipidemia, nicotine abuse and coronary artery disease. The vertical reference line represents an aOR of 1.0 (indicating no difference in risk)

Regarding safety outcomes, the adjusted odds for intracranial bleeding were highest in the mouth/throat group (aOR 1.64, 95%CI 0.50–5.34), while the female reproductive system group showed the lowest association (aOR 0.75, 95%CI 0.23–2.38). The association with acute anemia was massively pronounced in patients with gastrointestinal cancer (aOR 10.21, 95%CI 7.18–14.51) (Fig. 3).

In the thrombectomy cohort, respiratory cancer was associated with the highest adjusted odds of mortality (aOR 3.09, 95%CI 2.48–3.84), whereas patients with skin cancer (aOR 0.51, 95%CI 0.19–1.34) and breast cancer (aOR 0.77, 95%CI 0.49–1.21) showed the lowest risk. For hemorrhagic complications, urogenital cancer demonstrated the strongest association with intracranial bleeding (aOR 1.50, 95%CI 0.82–2.75). Notably, the risk for subarachnoid hemorrhage was highest among patients with prostate cancer (aOR 2.48, 95%CI 1.42–4.34). The likelihood of acute anemia was again highest in the gastrointestinal group (aOR 10.21, 95%CI 7.18–14.5).

**Fig. 4:**
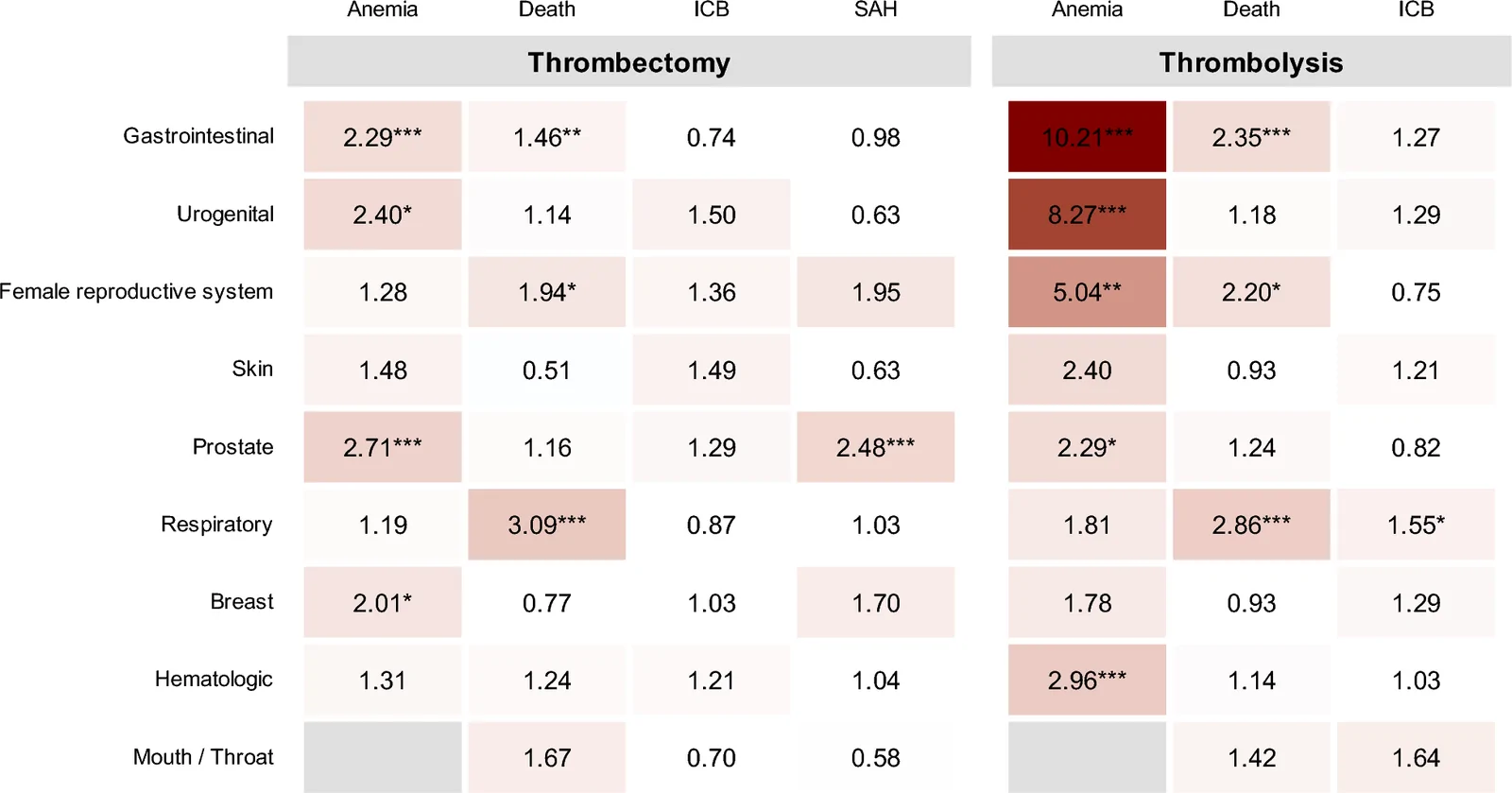
Heatmap of adjusted odds ratios (aOR) for major in-hospital complications by cancer subtype and recanalization therapy. Cell color indicates the magnitude of the adjusted risk compared to patients without cancer: blue indicates an aOR < 1.0 (lower odds), white indicates an aOR of 1.0 (equal odds), and red indicates an aOR > 1.0 (higher odds). Blank (gray) cells represent subgroups with zero events (complete separation) where adjusted estimates could not be reliably calculated and were consequently omitted. *ICB* intracranial bleeding; *SAH* subarachnoid hemorrhage; * = *p* < 0.05; ** = *p* < 0.01; *** = *p* < 0.001

To visualize the specific risk profiles, we generated a heatmap of adjusted odds ratios (aOR) for major complications compared to the control group (Fig. 4). While acute anemia risk was markedly elevated across multiple entities (especially gastrointestinal and urogenital), no single cancer subtype showed a statistically significant increase in the adjusted risk for general intracranial bleeding (ICB) following either intervention.

## Discussion

Here we present to our knowledge the largest nationwide analysis to provide data on the comparative safety across different cancer entities. Our data demonstrate that the risk of acute revascularization in stroke patients is highly heterogeneous and strongly dependent on the cancer subtype.

One of our primary findings is the association of two times higher in-hospital mortality in this cohort compared to non-cancer patients with different complication patterns in different cancer subtypes. These results are consistent with the findings of the largest registry study to date, involving over seven million patients, which reported a rate of fatal stroke of 21.64 per 100,000 person-years and a standardized mortality ratio (SMR) of fatal stroke of 2.17 (95% CI, 2.15, 2.19) [4]. An interesting finding was the lower prevalence of traditional cardiovascular risk factors (hypertension, hyperlipidemia, diabetes) in the EVT-cancer cohort. This strongly supports the concept of ‘cancer-associated stroke’ (CAS), where the stroke etiology is not related to cardiovascular risk factors but rather a cancer-specific mechanism. The pathophysiological concept of cancer-associated stroke is based on the idea of cancer-induced hypercoagulability (Trousseau syndrome) [10]. Interestingly, the South Korean ENCHASE study is currently investigating therapeutic anticoagulation based on this concept [11]. However, previous studies did not stratify risks for distinct cancer subtypes.

Our results align with trends in more specific substudies such as the MR CLEAN study, where significantly higher mortality and worse functional outcomes in cancer patients were reported. Our analysis complements this by demonstrating that the mortality risk is not uniform but driven by specific high-risk entities, particularly respiratory and gastrointestinal malignancies.

We identified gastrointestinal, respiratory, and female reproductive cancer as cancer subtypes with the highest risk type independent of acute therapy type. This aligns with the existing data on stroke risk in cancer patients, which also identified respiratory and gastrointestinal cancers as high-risk types. However, cancer of the female reproductive system was not among the high-risk cancers [12]. The largest populations of the cancer types identified here are adenocarcinomas (colon, lung, ovary), which fit perfectly with the mucin hypothesis of Trousseau syndrome, in which mucin is released from adenocarcinomas and reacts directly with P-selectin on thrombocytes, leading to massive platelet aggregation (white clots) [13]. The latter are associated with a poorer outcome [14]. In addition, non-bacterial endocarditis is a common complication in hypercoagulative states especially in ovarian cancer [15]. At the same time, those cancer subtypes are more often diagnosed in late stages [16, 17]. Interestingly, we observed a non-significant trend toward lower mortality in breast cancer patients following thrombectomy. This is biologically implausible as a protective effect and suggests a selection bias, wherein neurologists might selectively offer aggressive endovascular treatment only to the fittest patients with a history of breast cancer, possibly even in long-term remission.

A somewhat unexpected finding was that no cancer subtype showed a statistically significant increase in intracranial hemorrhage (ICH) risk after thrombolysis or thrombectomy. To visualize the specific risk profiles, we generated a heatmap of adjusted Odds Ratios (aOR) for major complications compared to the control group (Fig. 4). While acute anemia risk was markedly elevated across multiple entities (especially gastrointestinal and urogenital), no single cancer subtype showed a statistically significant increase in the adjusted risk for general intracranial bleeding (ICB) following either intervention. This apparent paradox is explained by the loss of statistical power upon stratifying the cohort into smaller subgroups, leading to widened confidence intervals. Nonetheless, our adjusted estimates indicate that the ICH risk appears manageable.

Our data powerfully challenge this historical fear of ICH in this cohort. This finding is consistent with recent study data on the risk of symptomatic ICH in thrombectomy and thrombolysis [8, 18, 19]. However, a possible explanation might be an underlying selection bias where patients with suspected intracranial neoplasia such as metastasis in CT-scans were excluded. Alternatively this fact might indicate the reliability of our inclusion criteria and existing in hospital workflow with high neuroradiologic skillsets. The specific, significant elevation of subarachnoid hemorrhage (SAH) following thrombectomy in prostate cancer patients is an intriguing finding. Mechanistically, advanced prostate cancer is notoriously associated with complex coagulopathies, including disseminated intravascular coagulation (DIC) in up to 25% of patients [20]. Upon mechanical reperfusion of fragile vasculature, this systemic hyperfibrinolysis might predispose these specific patients to vessel perforation or reperfusion bleeding presenting as SAH. Alternatively, the increase in SAH without a parallel increase in overall ICH might partially reflect a surveillance bias, as cancer patients often undergo more rigorous follow-up imaging.

Of note, the most frequent complication across all multiple subtypes was acute anemia (aOR > 10). Smaller cohort studies on stroke documented up to a third of all patients with at least mild anemia after intravenous thrombolysis or thrombectomy [21, 22]. Anemia in stroke is strongly associated with poor outcomes [23].

What are the clinical implications of our study? Our data support a decision to treat approach based on cancer subtype rather than cancer as a binary diagnosis. For patients with gastrointestinal or respiratory cancer, high vigilance and post-procedural monitoring for blood loss and the possibility for endoscopic treatment should be an obligation.

The primary strength of our study is the comprehensive nationwide case-based administrative dataset providing statistical power minimizing single-center report biases. However, we must acknowledge the inherent limitations of DRG-Data where coding-errors can occur and inaccuracies in recording cannot be ruled out. For example, OPS 8–800.0 not only records blood transfusions and red blood cell transfusions, but also platelet concentrates, and increased administration in hematological cancer patients could also be a symptom of the disease. Another limitation of this study is its retrospective approach, which allows for the identification of associations but precludes the establishment of definitive causal relationships. In-hospital mortality is a strong endpoint however we cannot distinguish between cancer related deaths and deaths due to acute therapy. Our analysis is case-based, meaning that if patients are readmitted for a recurrent stroke within the same year, they may be counted twice. This potential bias from higher recurrence rates in cancer patients leading to multiple counting remains a strict limitation of the study design. Although the number of patients requiring multiple acute revascularization therapies within a short timeframe is likely low. Furthermore, the lack of clinical severity parameters (e.g., NIHSS, ASPECTS) remains an unmeasured confounder, although we rigorously adjusted our multivariable models for age, sex, and cardiovascular comorbidities. The inability to differentiate between active and inactive malignancies (as ICD-10 coding does not reliably distinguish between active, metastatic, and remote cancer) likely dilutes the true magnitude of risk, as existing literature demonstrates that elevated stroke mortality is primarily driven by active and metastatic disease [24]. Unlike the ITACA trial⁸, we did not include a control group of cancer patients with stroke who received best medical management only. Therefore, we cannot draw conclusions regarding the efficacy of revascularization compared to best medical treatment but rather provide data on the comparative safety across different cancer entities.

In conclusion, our nationwide analysis of 193,863 cases implies that the risk of acute revascularization in stroke patients is highly heterogeneous and dependent on cancer subtype. We provide evidence that complements existing clinical case series. While mortality and specific bleeding risks are generally elevated in stroke patients with malignancies, these risks depend strongly on the cancer subtype. However, we acknowledge that clinically meaningful increases in ICH risk for specific subtypes might be obscured by wide confidence intervals due to limited sample sizes. Systemic bleeding complications, particularly anemia in gastrointestinal malignancies, emerged as a key risk factor in our analysis. These findings advocate for a tailored, risk-based approach to recanalization therapies, weighing individual complication profiles. This approach aims to optimize patient safety and management, potentially improving outcomes in this vulnerable population, rather than adhering to a general exclusion of cancer patients from acute stroke therapy.

## Supplementary Information

Below is the link to the electronic supplementary material.

Supplementary file1 (PDF 85 KB)

Supplementary file2 (PDF 102 KB)

## Data Availability

The datasets used and/or analyzed during the current study are available from the corresponding author on reasonable request.
