# Supplementary material for: Recanalization therapy in stroke patients with malignancies: in-hospital outcomes by cancer subtype in a nationwide administrative data analysis
Source: J Neurol. 2026 May 29;273(6):347. doi: 10.1007/s00415-026-13851-9 (PMC13221337; doi:10.1007/s00415-026-13851-9)
Supplement: Supplementary file 1 — Supplementary file1 (PDF 85 KB) [file 415_2026_13851_MOESM1_ESM.pdf]

# RECORD Checklist (Extension for Routine Health Data)

| RECORD Item | Statement | Location in manuscript (Section / Paragraph) |
|---|---|---|
| **RECORD 1.1** | The type of data used should be specified in the title or abstract. | Title ("administrative data analysis"); Abstract (Methods section) |
| **RECORD 1.2** | Geographic region and timeframe within which the study took place should be reported. | Title ("nationwide"); Abstract (Methods: "in Germany") |
| **RECORD 1.3** | If linkage between databases was conducted, this should be clearly stated. | N/A (No linkage between different databases) |
| **RECORD 6.1** | The methods of study population selection (such as codes or algorithms) should be listed. | Methods, Study population (Paragraph 2: exact ICD-10 & OPS codes provided) |
| **RECORD 6.2** | Any validation studies of the codes or algorithms used should be referenced. | Methods, Study population (Paragraph 1: "rigorous external review by insurance companies (MDK)") |
| **RECORD 6.3** | If linkage of databases, consider use of a flow diagram. | N/A (Single database, but selection flow is provided in Figure 1) |
| **RECORD 7.1** | A complete list of codes and algorithms used to classify exposures, outcomes, etc. | Methods, Study population (Paragraph 2); Results, Outcome of patients... (ICD & OPS codes in text) |
| **RECORD 12.1** | Extent to which the investigators had access to the database population. | Methods, Study population (Paragraph 2: "Admission, use and data security is closely supervised...") |
| **RECORD 12.2** | Information on the data cleaning methods used in the study. | Methods, Study population (Paragraph 2: handling of missed values due to data |

| | | |
|---|---|---|
| | | protection rules) |
| **RECORD 12.3** | State whether the study included person-level, institutional-level, or other data linkage. | Methods |
| **RECORD 13.1** | Describe in detail the selection of the persons included (including filtering). | Figure 1; Methods, Study population (Paragraph 2) |
| **RECORD 19.1** | Discuss the implications of using data that were not created to answer the research question(s). | Discussion (Limitations, Paragraphs 5 & 6: coding errors, missing NIHSS/mRS) |
| **RECORD 22.1** | Information on how to access any supplemental information (protocol, raw data, code). | Declarations (Availability of data) |
