# Supplementary material for: Recanalization therapy in stroke patients with malignancies: in-hospital outcomes by cancer subtype in a nationwide administrative data analysis
Source: J Neurol. 2026 May 29;273(6):347. doi: 10.1007/s00415-026-13851-9 (PMC13221337; doi:10.1007/s00415-026-13851-9)
Supplement: Supplementary file 2 — Supplementary file2 (PDF 102 KB) [file 415_2026_13851_MOESM2_ESM.pdf]

STROBE Checklist (Cohort Studies)

Item No	Recommendation	Location in manuscript (Section / Paragraph)
**1a**	Indicate the study's design with a commonly used term in the title or the abstract	Title; Abstract (Methods section)
**1b**	Provide in the abstract an informative and balanced summary of what was done and what was found	Abstract (Complete)
**2**	Explain the scientific background and rationale for the investigation being reported	Introduction (Paragraphs 1-3)
**3**	State specific objectives, including any prespecified hypotheses	Introduction (Last paragraph: "The objective of this study...")
**4**	Present key elements of study design early in the paper	Methods, Study population (Paragraph 1)
**5**	Describe the setting, locations, and relevant dates, including periods of recruitment, exposure, follow-up, and data collection	Methods, Study population (Paragraph 1)
**6a**	Give the eligibility criteria, and the sources and methods of selection of participants. Describe methods of follow-up	Methods, Study population (Paragraphs 1 & 2); Figure 1
**6b**	For matched studies, give matching criteria and number of exposed and unexposed	N/A (No matching applied)
**7**	Clearly define all outcomes, exposures, predictors, potential confounders, and effect modifiers. Give diagnostic criteria, if applicable	Methods, Study population (Paragraph 2); Methods, Statistical Analysis (Paragraph 2)
**8**	For each variable of interest, give sources of data and details of methods of assessment (measurement)	Methods, Study population (Paragraphs 1 & 2)
**9**	Describe any efforts to address potential sources of bias	Discussion (Limitations, Paragraphs 5 & 6)

**10**	Explain how the study size was arrived at	Methods, Study population; Figure 1
**11**	Explain how quantitative variables were handled in the analyses. If applicable, describe which groupings were chosen and why	Methods, Statistical Analysis (Paragraphs 1 & 2)
**12a**	Describe all statistical methods, including those used to control for confounding	Methods, Statistical Analysis (Paragraphs 1 & 2)
**12b**	Describe any methods used to examine subgroups and interactions	Methods, Statistical Analysis (Paragraph 2: "...association between specific cancer subtypes...")
**12c**	Explain how missing data were addressed	Methods, Study population (Paragraph 2: "...missed value due to data protection rules.")
**12d**	If applicable, explain how loss to follow-up was addressed	N/A (Cross-sectional in-hospital outcome only)
**12e**	Describe any sensitivity analyses	Not conducted
**13a**	Report numbers of individuals at each stage of study	Results, Baseline characteristics (Paragraph 1); Figure 1
**13b**	Give reasons for non-participation at each stage	Figure 1
**13c**	Consider use of a flow diagram	Figure 1
**14a**	Give characteristics of study participants (eg demographic, clinical, social)	Results, Baseline characteristics (Paragraph 1); Table 1
**14b**	Indicate number of participants with missing data for each variable of interest	Methods, Study population (Paragraph 2)
**14c**	Summarise follow-up time (eg, average and total amount)	N/A (Cross-sectional in-hospital outcome only)
**15**	Report numbers of outcome events or	Results, Outcome of patients

	summary measures over time	with acute therapy... (Paragraphs 1 & 2); Tables 2 & 3
**16a**	Give unadjusted estimates and, if applicable, confounder-adjusted estimates...	Results, Outcome of patients with acute therapy and cancer localization; Figures 2, 3, 4
**16b**	Report category boundaries when continuous variables were categorized	Not applicable
**16c**	If relevant, consider translating estimates of relative risk into absolute risk...	Results, Outcome of patients... (Paragraph 3 / Figure 4: Relative Risks)
**17**	Report other analyses done—eg analyses of subgroups and interactions...	Results, Outcome of patients with acute therapy and cancer localization (Stratification by cancer subtype)
**18**	Summarise key results with reference to study objectives	Discussion (Paragraphs 1 & 2)
**19**	Discuss limitations of the study, taking into account sources of potential bias...	Discussion (Limitations, Paragraphs 5 & 6)
**20**	Give a cautious overall interpretation of results considering objectives...	Discussion (Paragraphs 1-4 & 7: Conclusion)
**21**	Discuss the generalisability (external validity) of the study results	Discussion (Paragraph 5: Nationwide dataset; Paragraph 7: Conclusion)
**22**	Give the source of funding and the role of the funders for the present study	Declarations (Funding)
